# Vestibular Function After the 2016 Kumamoto Earthquakes: A Retrospective Chart Review

**DOI:** 10.3389/fneur.2020.626613

**Published:** 2021-01-22

**Authors:** Toru Miwa

**Affiliations:** ^1^Department of Otolaryngology and Head and Neck Surgery, Graduate School of Medicine, Kyoto University, Kyoto, Japan; ^2^Department of Otolaryngology and Head and Neck Surgery, Kitano Hospital, Tazuke Kofukai Medical Research Institute, Osaka, Japan; ^3^Department of Otolaryngology and Head and Neck Surgery, Graduate School of Medicine, Kumamoto University, Kumamoto, Japan

**Keywords:** dizziness, earthquake, equilibrium, psychological stress, vertigo, vestibular function

## Abstract

This retrospective chart review aimed to examine both the vestibular function and causes of dizziness experienced by individuals following a series of major earthquakes and repetitive aftershocks. All patients with balance disorders who experienced the 2016 Kumamoto earthquakes and their aftershocks completed questionnaires relevant to balance disorders and were enrolled in this study after providing informed consent. There were 2.8 times more patients with balance disorders post the earthquake. Anxiety (*P* = 0.02), orthostatic dysregulation (*P* = 0.005), and motion sickness scores (*P* = 0.03) were all significantly higher after the earthquakes. A subset of participants underwent clinical equilibrium testing, showing significant deteriorations in the equilibrium test results (stabilometry: *P* = 0.01), cervical vestibular-evoked myogenic potentials (*P* = 0.04), and head-up tilt (*P* = 0.03) after the earthquake. The findings of this study also suggest that earthquake-induced disequilibrium may be further influenced by physical stressors, including sensory disruptions induced by earthquake vibrations, changes in the living conditions, and autonomic stress. This study increases our understanding of human equilibrium in response to natural disasters. Moreover, these findings will facilitate the management of dizziness experienced during or after such disasters. Future studies should identify strategies for mitigating autonomic dysfunction to prevent post-earthquake dizziness.

## Introduction

Major earthquakes are associated with an increased prevalence of psychiatric morbidities (1, 2), sleep disorders (3, 4), and dizziness (5–7). The Kumamoto earthquakes, which occurred on April 14 and 16, 2016, (moment magnitude = 9.0) and included several high-magnitude vibrations and aftershocks without secondary disasters, led to reportedly significant outbreaks of dizziness over a large area surrounding the earthquake epicenter several months after the initial earthquake. Such an occurrence is not unusual after a major earthquake (8).

Although several reports have described post-earthquake dizziness (5–7), the characteristic symptoms of dizziness remain undefined. After the Tohoku earthquake on March 11, 2011 (moment magnitude = 9.0) and its sequelae (a tsunami and the Fukushima nuclear disaster), Nomura et al. defined the characteristic symptoms of post-earthquake dizziness as post-earthquake dizziness syndrome (PEDS) (9). Individuals without balance disorders but with PEDS experienced illusory body swaying lasting <1 min within 3 months after the earthquake. PEDS is reportedly caused by psychological stress and mismatched visual/somatosensory inputs that induce autonomic dysfunction (9). Although studies have reported vertigo in several individuals with PEDS after the Tohoku earthquake, the influence of major earthquakes and repetitive aftershocks on the onset or recurrence of balance disorders remains unexamined. Therefore, this study aimed to examine how major earthquakes and repetitive aftershocks influence the human equilibrium system. I hypothesized that (1) the prevalence of earthquake-related dizziness (ERD) increases after major earthquakes and repetitive aftershocks; and (2) that ERD is caused by functional vestibular disturbances and autonomic dysfunction, mediated by physiological and psychological stressors.

## Materials and Methods

### Participants

The patient records were reviewed between and data collected from 21 otolaryngology clinics and hospitals in Kumamoto prefecture April 1st and July 21st, 2016 were extracted. Data were analyzed between 2017 and 2019. Inclusion criteria were a cumulative total patients with new-onset and worsened balance disorders, who visited otolaryngology clinics or hospitals in Kumamoto prefecture between April 1st and July 21st, 2016, were aged between 10 and 100 years, and could describe their symptoms and experiences following the Kumamoto earthquakes. The exclusion criterion was individuals who had not visited an otolaryngology clinic or hospital due to balance disorders. Participants who reported vertigo/dizziness before major earthquakes and visited otolaryngology clinic or hospital after major earthquakes were classified as experiencing balance disorders in the pre-earthquake period.

The study adhered to the tenets of the Declaration of Helsinki and was approved by the Institutional Ethics Committee of Kumamoto University Hospital (approval number: 1099). All participants enrolled in the study provided written informed consent.

### Main Measures and Outcomes

Data, including sex, age, symptoms, history of vertigo/dizziness, floor location during the earthquake, evacuation methods, and diagnosis, which were obtained on the day of ear, nose, and throat (ENT) consultation between April 1st and July 21st, 2016, were collected from hospital/clinic medical records and analyzed. Clinical diagnosis of balance disorders was based on the diagnostic criteria published by the Japan society for equilibrium research (10, 11). All participants completed both clinical questionnaire-based surveys regarding balance disorders and a series of equilibrium tests after the major earthquakes (April 14th to July 21st) and the results were then compared to those before the earthquake (April 1st to 13th). Based on the earthquake magnitude database, no aftershocks were recorded during the examination period. The clinical questionnaires administered were the Dizziness Handicap Inventory (DHI) (12, 13); Hospital Anxiety and Depression Scale (HADS) (14, 15); and orthostatic dysregulation (OD) questionnaires (16, 17), Graybiel's motion sickness test (18, 19), and the Epworth Sleepiness Scale (20, 21). Examinations included equilibrium tests for Romberg ratios (ratio of closed/open area or length of sway) in stabilometry to assess steady-state postural control (22); cervical vestibular-evoked myogenic potential (cVEMP) testing to assess the function of the saccule-inferior vestibular nerve system (23); caloric testing to assess the function of the utricle-superior vestibular nerve system (24); head-up tilt (HUT) testing to assess OD, which is related to autonomic dysfunction, especially that of the sympathetic nervous system (25); and nystagmus to assess vestibular function.

Methodological details and criteria regarding the questionnaires and tests are described in Supplementary Table 1. In brief, during stabilometry, the patients stood on a strain-gauge force platform (GP-31 stabilometer; Anima, Tokyo, Japan) for 60 s with their eyes first open and then closed. Measurements were performed under background noise conditions (~50 dB). The area and total length of body sway were measured, and the Romberg ratio (RR) was calculated. RR was defined as the body sway area or length with the eyes closed divided by the same parameter with the eyes open. An RR-area of >1.29 and an RR-length of >1.22 were considered to represent stability deterioration (26). During the cVEMP test, the patient's neck was rotated to the left side, as far as possible (~70°-80°). The stimulation utilized clicks with 120 dB sound pressure level lasting 0.1 ms, with a stimulation frequency of 5 Hz and an analysis time of 50 ms. The electromyographic responses to 200 stimuli were averaged and recorded using an evoked potential recorder with a band-pass filter of 20–2000 Hz (Neuropack; Nihon Kohden, Tokyo, Japan). To assess cVEMP amplitude, the asymmetry ratio (AR) was used, which was defined as the difference between the large amplitude (AL) and small amplitude (AS) of peak 13 to peak n23 divided by the sum of both amplitudes presented as a percent, i.e., [(AL-AS)/(AL+AS)] × 100(%). The normal range of the AR was defined as <33% (27). During the caloric test, stimulation was provided through sequential irrigation of each ear with 20 mL of warm or cold water for 10 s. The maximum slow-phase velocity (MVS) was measured using videonystagmography recordings (Meditester VOG CD8001, Panasonic, Osaka, Japan). To assess caloric test, canal paresis % (CP%) was calculated using the following equation: [[(MVS of the right warm (RW)+ MVS of the right cold (RC))-(MVS of the left warm (LW)+ MVS of the left cold (LC))]/(RW+RC+LW+LC)] × 100(%).

The normal range of the CP% was defined as <20% (28). The HUT test was performed according to the method established by the Japan Society of Neurovegetative Research in 2015 (29). Non-invasive oscillatory measurements of blood pressure (BP) and pulse rate were performed four times using an automated sphygmomanometer (ES-H55P; Terumo, Tokyo, Japan) at the following time points: (1) after 10 min in a supine position, (2) after 10 s of standing, (3) after 1 min of standing, and (4) after 10 min of standing (29). The cuff of the BP-recording device was attached to the left arm, which was supported at the heart level throughout the study. The testing was conducted during the daytime in a quiet environment at a constant room temperature of 22–25°C to exclude the effects of chronobiologic factors on the outcomes of the test. The participants maintained a regular meal schedule but were restricted from smoking and caffeine ingestion for 6 h before the examination. The intake of foods and medications with sympathomimetic activity was also prohibited before the study. The results were determined as positive or negative according to the outcome of the HUT test and the international scientific definition of OD (Supplementary Table 1) (30). Nystagmus was evaluated by the infrared CCD camera. When pathologic nystagmus (i.e., spontaneous nystagmus or positional nystagmus) was observed, the test result was considered positive.

The primary outcome was the occurrence of post-earthquake dizziness (i.e., vertigo/dizziness experienced by participants after an earthquake), and the secondary outcomes were psychological stress and vestibular and autonomic dysfunction.

### Statistical Analyses

Power and sample size calculations were conducted before and after data collection using the PS software (Ver. 3.1.6, Vanderbilt University, Nashville, TN) (31). Regarding the primary outcome, changes in the proportion of patients with balance disorders before and after the earthquake were compared using one-way analysis of variance (ANOVA) and *post hoc* Dunnett's test to avoid an inflated Type I error rate because data were normally distributed. Regarding secondary outcomes, since the data were also normally distributed, both one-way ANOVA and *post hoc* Tukey–Kramer tests were used to investigate the ratio of patients with ERD who were evacuated to a shelter to avoid an inflated Type I error rate. Residual plots were used to confirm the correctness of the assumptions made for both primary and secondary outcomes. Missing values were imputed by random forest method. There were no outliers in the analysis of either the primary or the secondary outcomes. For non-parametric analysis of subjective variables, which were not normally distributed, Wilcoxon signed-rank tests were used to investigate changes in the pre- and post-earthquake questionnaire scores and the equilibrium test results in patients with ERD who underwent equilibrium testing before and after the earthquake. Fisher's exact test was used to compare pre- and post-earthquake HUT and nystagmus scores with an abnormal distribution in patients who underwent equilibrium testing before and after the earthquake. Bland-Altman analysis with test-retest intervals was performed to discern test-retest effects from clinical changes. Statistical significance was set at *P* < 0.05. Evaluations were determined as “not applicable” if the calculated sample size after data collection was found to be insufficient for statistical analysis. All statistical analyses were performed with EZR (Saitama Medical Center, Jichi Medical University, Saitama, Japan), which is a graphical user interface for R (The R Foundation for Statistical Computing, Vienna, Austria) and a modified version of R commander designed to add statistical functions frequently used in biostatistics.

## Results

### Prevalence of ERD After the Kumamoto Earthquakes

**Figure 1:**
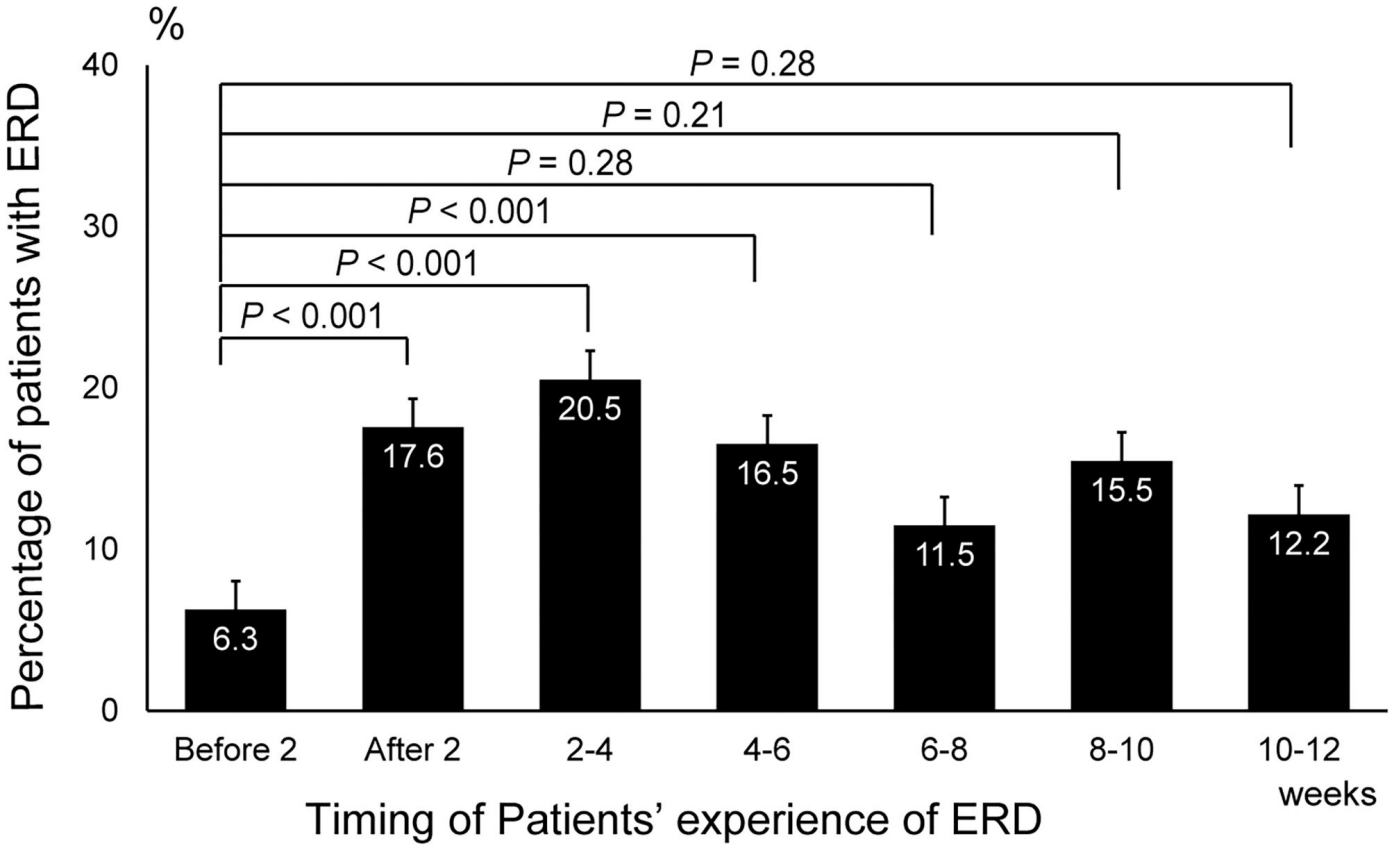
Changes in the prevalence of earthquake-related dizziness before and after the earthquake. The number of patients who experienced earthquake-related dizziness during the 2-week, 2–4-week, and 4–6-week post-earthquake periods was significantly higher than that during the 2-week period before the earthquake (*P* < 0.001, one-way ANOVA and *post hoc* Dunnett's test). ERD, earthquake-related dizziness.

A cumulative total of 575 patients (female, 424; male, 151) diagnosed with balance disorders after the Kumamoto earthquakes participated in this study. The median (interquartile range) age in this cohort was 63.5 (16-93) years in men and 59.0 (10-95) years in women. The proportion of patients with balance disorders was significantly higher during the 0–2-week (17.5, 95% confidence interval [CI]: 10.6–26.4), 2–4-week (20.5, 95% CI: 13.6–28.9), and 4–6-week (16.5, 95% CI: 9.64–25.5) post-earthquake periods than the 2-week pre-earthquake period (6.26, 95% CI: 0.77–20.0) (all *P* < 0.001; Figure 1). Detailed information on the frequency of ERD according to sex, age, symptoms, and location at different time points relative to the earthquake is presented in Supplementary Tables 2–4. The complete list of balance disorders diagnosed after the earthquake by 21 ENT doctors is presented in Supplementary Table 5.

Significantly higher proportions of patients were diagnosed with Meniere's disease (MD; 17.9, 95% CI: 6.60–28.5, *P* = 0.02), sudden deafness with vertigo (SDV; 19.5, 95% CI: 6.00–33.0, *P* = 0.01), vertebrobasilar insufficiency (VBI; 27.0, 95% CI: 6.01–47.9, *P* = 0.01), psychogenic vertigo (21.0, 95% CI: 6.04–35.9, *P* = 0.003), and vestibular neuritis (VN; 12.0, 95% CI: 10.1–34.1, *P* = 0.04) during the post-earthquake period than during the pre-earthquake period (Table 1). The proportion of patients diagnosed with benign paroxysmal positional vertigo (BPPV) and vertigo or dizziness due to unstable blood pressure (so called orthostatic hypotension (OH)) was significantly higher during the 2–4-week post-earthquake period than during the pre-earthquake period (BPPV: 19.0, 95% CI: 4.28–33.7, *P* = 0.01; OH: 19.0, 95% CI: 4.28–33.7, *P* = 0.003; Table 1).

**Table 1:** Prevalence of balance disorders relative to the time of earthquake in weeks (w).

	**MD (%)**	**SDV (%)**	**BPPV (%)**	**VBI (%)**	**OH (%)**	**Psychogenic vertigo (%)**	**VN (%)**
2 w prior	4.4 ± 2.4	2.4 ± 2.4	7.1 ± 2.0	4.5 ± 4.4	8.3 ± 3.9	8.3 ± 3.9	6.3 ± 6.0
After 2 w^a^	22.1 ± 5.0 (*P* = 0.02)	22.0 ± 6.4(*P* = 0.01)	7.1 ± 2.0 (*P* = 0.51)	31.8 ± 9.9(*P* = 0.01)	16.7 ± 5.3 (*P* = 0.18)	29.2 ± 6.5(*P* = 0.003)	18.8 ± 9.7 (*P* = 0.04)
After 2–4 w^a^	26.5 ± 5.3 (*P* = 0.001)	14.6 ± 5.5(*P* = 0.01)	18.1 ± 3.0 (*P* = 0.01)	13.6 ± 7.3(*P* = 0.22)	27.1 ± 6.4 *(P* =0.003)	25.0 ± 6.2(*P* = 0.04)	12.5 ± 8.2 (*P* = 0.67)
After 4–6 w^a^	13.2 ± 4.1 (*P* = 0.06)	12.2 ± 5.1(*P* = 0.02)	16.8 ± 3.0 (*P* = 0.01)	31.8 ± 9.9(*P* = 0.01)	14.6 ± 5.0 (*P* = 0.18)	12.5 ± 4.7(*P* = 0.56)	12.5 ± 8.2 (*P* = 0.67)
After 6–8 w^a^	13.2 ± 4.1 (*P* = 0.06)	14.6 ± 5.5(*P* = 0.03)	16.8 ± 3.0 (*P* = 0.01)	18.2 ± 8.2(*P* = 0.22)	16.7 ± 5.3 (*P* = 0.18)	10.4 ± 4.4(*P* = 0.56)	12.5 ± 8.2 (*P* = 0.77)
After 8–10 w^a^	11.8 ± 3.9 (*P* = 0.36)	17.1 ± 5.8(*P* = 0.02)	18.1 ± 3.0 (*P* = 0.01)	0.0 ± 0.0(*P* = 0.78)	8.3 ± 3.9 (*P* = 0.56)	8.3 ± 3.9(*P* = 0.66)	18.8 ± 9.7 (*P* = 0.76)
After 10–12 w^a^	8.8 ± 3.4 (*P* = 0.36)	17.1 ± 5.8(*P* = 0.02)	16.1 ± 2.9 (*P* = 0.01)	0.0 ± 0.0(*P* = 0.79)	8.3 ± 3.9 (*P* = 0.56)	6.3 ± 3.4(*P* = 0.66)	18.8 ± 9.7 (*P* = 0.26)

a *All P-values were calculated using ANOVA and the post hoc Dunnett's test*.

*MD, Meniere disease; SDV, sudden deafness with vertigo; VBI, vertebrobasilar insufficiency; BPPV, benign paroxysmal positional vertigo; OH, orthostatic hypotension; VN, vestibular neuritis*.

### Causes of ERD

To determine whether earthquakes influenced balance disorders in the 575 patients enrolled in the study, I assessed their medical histories for vertigo/dizziness. When restricting analysis to patients with no history of balance disorders, I observed that a greater proportion of patients experienced SDV (19.4, 95% CI: 5.96%−32.9%, *P* = 0.01), VBI (16.6, 95% CI: 1.71–31.4, *P* < 0.001), OH (7.04, 95% CI: 6.39–19.82, *P* = 0.03), or psychogenic vertigo (15.3, 95% CI: 0.94–29.8, *P* = 0.01) during the 0–2-week post-earthquake period than during the 2-week pre-earthquake period (Table 2). The proportion of patients experiencing MD or BPPV was greater during the 2–4-week post-earthquake period than during the pre-earthquake period (MD: 8.82, 95% CI: 0.18–17.%, *P* = 0.03; BPPV: 8.67, 95% CI: 3.53–13.8, *P* = 0.04; Table 2); moreover, the proportion of patients with MD and VBI was greater during the 0–2-week post-earthquake period than during the 2-week pre-earthquake period (MD: 16.1, 95% CI: 6.67–25.6, *P* = 0.004; VBI: 12.5, 95% CI: 0.73–25.7, *P* = 0.03; Table 2), and the proportion of patients experiencing BPPV, OH, and VN was greater during the 2–4-week post-earthquake period than during the pre-earthquake period (BPPV: 5.33, 95% CI: 0.94–9.73, *P* = 0.04; OH: 6.32, 95% CI: 5.49–18.13, *P* = 0.03; VN: 12.5, 95% CI: 3.70–28.7, *P* < 0.001; Table 2).

**Table 2:** Prevalence of balance disorders in relation to a history of balance disorder relative to the time of the earthquake in weeks (w).

		**MD** **(%)**	**SDV** **(%)**	**BPPV** **(%)**	**VBI** **(%)**	**OH** **(%)**	**Psychogenic vertigo** **(%)**	**VN** **(%)**
No history of vestibular diseases	2 w prior	2.9 ± 2.0	2.4 ± 2.4	1.3 ± 0.9	0.0 ± 0.0	2.1 ± 2.1	5.1 ± 3.5	6.3 ± 6.0
	After 2 w	4.4 ± 2.4 (*P* = 0.26)	22.0 ± 6.4 (*P* = 0.03)	4.0 ± 1.6 (*P* = 0.51)	16.7 ± 7.6 (*P* < 0.001)	8.5 ± 4.0 (*P* = 0.03)	20.5 ± 6.4 (*P* = 0.01)	12.5 ± 8.2 (*P* = 0.65)
	After 2–4 w	11.8 ± 3.9 (*P* = 0.03)	14.6 ± 5.5 (*P* = 0.04)	10.0 ± 2.4 (*P* = 0.04)	4.2 ± 4.0 (*P* = 0.45)	14.9 ± 5.1 (*P* = 0.03)	12.8 ± 5.3 (*P* = 0.23)	0.0 ± 0.0 (*P* = 0.87)
	After 4–6 w	0.0 ± 0.0 (*P* = 0.98)	12.2 ± 5.1 (*P* = 0.04)	10.7 ± 2.5 (*P* = 0.04)	25.0 ± 8.8 (*P* < 0.001)	10.6 ± 4.4 (*P* < 0.001)	12.8 ± 5.3 (*P* = 0.23)	6.3 ± 6.0 (*P* = 0.99)
	After 6–8 w	4.4 ± 2.4 (*P* = 0.86)	14.6 ± 5.5 (*P* = 0.04)	10.7 ± 2.5 (*P* = 0.04)	0.0 ± 0.0 (*P* = 0.98)	2.1 ± 2.1 (*P* = 0.67)	5.1 ± 3.5 (*P* = 0.78)	6.3 ± 6.0 (*P* = 0.99)
	After 8–10 w	2.9 ± 2.0 (*P* = 0.86)	17.1 ± 5.8 (*P* = 0.05)	10.7 ± 2.5 (*P* = 0.04)	8.3 ± 5.6 (*P* = 0.28)	2.1 ± 2.1 (*P* = 0.67)	0.0 ± 0.0 (*P* = 0.97)	12.5 ± 8.2 (*P* = 0.65)
	After 10–12 w	7.4 ± 3.1 (*P* = 0.89)	17.1 ± 5.8 (*P* = 0.05)	11.3 ± 2.5 (*P* = 0.04)	0.0 ± 0.0 (*P* = 0.98)	4.3 ± 2.9 (*P* = 0.43)	7.7 ± 4.2 (*P* = 0.76)	12.5 ± 8.2 (*P* = 0.65)
History of vestibular diseases	2 w prior	1.5 ± 1.4	NA	1.3 ± 0.9	4.2 ± 4.0	6.4 ± 3.5	5.1 ± 3.5	0.0 ± 0.0
	After 2 w	17.6 ± 4.6 (*P* = 0.004)	NA	3.3 ± 1.4 (*P* = 0.65)	12.5 ± 6.7 (*P* = 0.03)	8.5 ± 4.0 (*P* = 0.36)	7.7 ± 4.2 (*P* = 0.34)	6.3 ± 6.0 (*P* = 0.23)
	After 2–4 w	14.7 ± 4.2 (*P* = 0.09)	NA	6.7 ± 2.0 (*P* = 0.04)	8.3 ± 5.6 (*P* = 0.45)	12.8 ± 4.8 (*P* = 0.03)	10.3 ± 4.8 (*P* = 0.22)	12.5 ± 8.2 (*P* < 0.001)
	After 4–6 w	11.8 ± 3.9 (*P* = 0.09)	NA	6.0 ± 1.9 (*P* = 0.09)	4.2 ± 4.0 (*P* = 0.76)	4.3 ± 2.9 (*P* = 0.67)	0.0 ± 0.0 (*P* = 0.98)	6.3 ± 6.0 (*P* = 0.23)
	After 6–8 w	5.9 ± 2.8 (*P* = 0.25)	NA	6.0 ± 1.9 (*P* = 0.09)	8.3 ± 5.6 (*P* = 0.45)	4.3 ± 2.9 (*P* = 0.76)	2.6 ± 2.5 (*P* = 0.89)	6.3 ± 6.0 (*P* = 0.23)
	After 8–10 w	13.2 ± 4.1 (*P* = 0.09)	NA	5.3 ± 1.8 (*P* = 0.10)	8.3 ± 5.6 (*P* = 0.45)	14.9 ± 5.1 (*P* = 0.02)	10.3 ± 4.8 (*P* = 0.22)	6.3 ± 6.0 (*P* = 0.23)
	After 10–12 w	2.9 ± 2.0 (*P* = 0.87)	NA	6.7 ± 2.0 (*P* = 0.04)	0.0 ± 0.0 (*P* = 0.98)	4.3 ± 2.9 (*P* = 0.76)	0.0 ± 0.0 (*P* = 0.98)	6.3 ± 6.0 (*P* = 0.23)

*All P-values were calculated using ANOVA and post hoc Dunnett's test*.

*MD, Meniere's disease; SDV, sudden deafness with vertigo; VBI, vertebrobasilar insufficiency; BPPV, benign paroxysmal positional vertigo; OH, orthostatic hypotension; VN, vestibular neuritis; NA, not applicable*.

**Figure 2:**
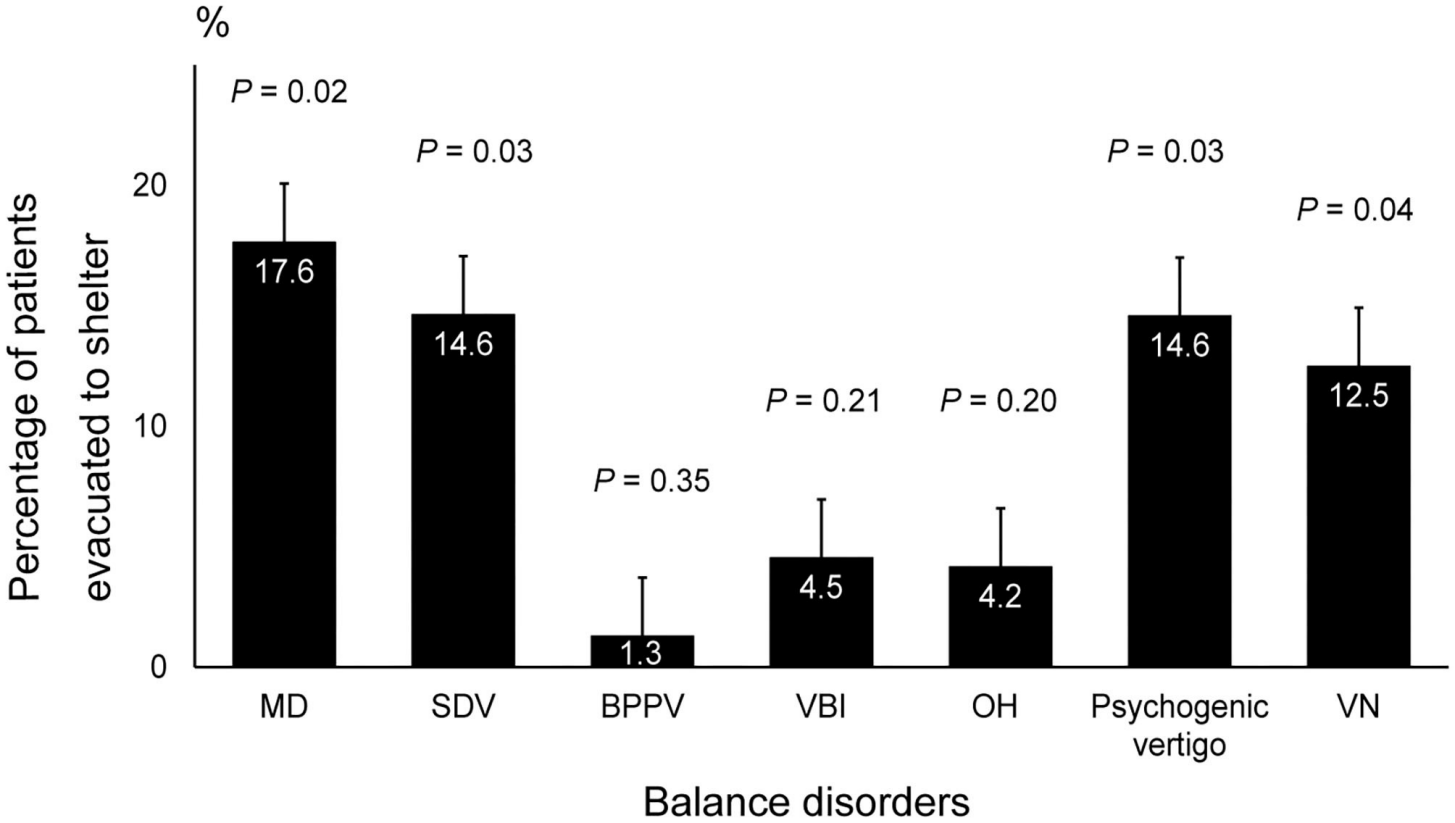
Rate of shelter evacuation in patients with balance disorders. The incidence of MD, SDV, psychogenic vertigo, and VN was significantly higher among patients who were evacuated to a shelter compared to that of the other balance disorders (*P* = 0.02, *P* = 0.03, *P* = 0.03 and *P* = 0.04, respectively, ANOVA and *post hoc* Tukey–Kramer test). MD, Meniere's disease; SDV, sudden deafness with vertigo; VN, vestibular neuritis; ANOVA, analysis of variance.

The results of the questionnaire-based survey showed significantly higher evacuation rates from cars or shelters in patients with MD (17.5, 95% CI: 8.55–26.6, *P* = 0.02), SDV (14.6, 95% CI: 3.79–25.41, *P* = 0.03), psychogenic vertigo (14.5, 95% CI: 4.57–24.5, *P* = 0.03), and VN (12.5, 95% CI: 0.00–28.7, *P* = 0.04) than in those with other balance disorders (Figure 2).

Among the 41 participants who underwent equilibrium testing before and after the earthquake, I found higher overall DHI scores (9.90, 95% CI: 5.84–25.6, *P* = 0.003) and DHI sub-scores for physical (5.18, 95% CI: 1.56–8.79, *P* = 0.01), emotional (0.63, 95% CI: 0.46–7.73, *P* = 0.04), and functional (3.00, 95% CI: 2.26–10.2, *P* = 0.02) subscales after the earthquake compared to those before the earthquake. Similarly, the scores for patient anxiety (2.18, 95% CI: 0.06–4.30, *P* = 0.02) and depression (5.63, 95% CI: 0.61–10.6, *P* = 0.03) components of HADS, OD (1.36, 95% CI: 0.35–2.37, *P* = 0.005), motion sickness (7.45, 95% CI: 2.27–17.1, *P* = 0.03), and ESS (0.82, 95% CI: 0.90-1.45, *P* = 0.09) were found to be higher after the earthquake than those before (Table 3). Analysis of stabilometry measurements revealed that RRs were significantly higher after the earthquake compared to those before the earthquake (areas: 0.47, 95% CI: 0.23–0.94, *P* = 0.01; lengths: 0.49, 95% CI: 0.11–1.09, *P* = 0.04; Table 4). AR of cVEMPs was also smaller after the earthquake than that before the earthquake (−21.7, 95% CI: −22.0−7.38, *P* < 0.001; Table 4). Conversely, no significant differences were found between the pre- and post-earthquake CP% of caloric responses (−2.97, 95% CI: −2.91–2.80-, *P* = 0.17) (Table 4). The rates of positive head-up tilt (HUT) test results were significantly higher after the earthquake compared to those before the earthquake, including the results of OD parameters (34.0, 95% CI: 3.60–64.4, *P* = 0.03) and positive rate of nystagmus (73.6, 95% CI: 58.3–88.8, *P* < 0.001) (Table 4).

**Table 3:** Results of the Clinical questionnaire-based survey before and after the earthquake.

**Questionnaire**		***n***	**Scores (pre)**	**Scores (post)**	***P*-value^a^**
DHI	Total	41	36.1 ± 20.2	41.7 ± 22.4	*P* = 0.003
	Physical	41	9.0 ± 6.1	12.9 ± 6.1	*P* = 0.01
	Emotional	41	13.5 ± 9.3	15.3 ± 8.7	*P* = 0.04
	Functional	41	14.4 ± 8.6	15.5 ± 10.2	*P* = 0.02
HADS	Anxiety	41	6.4 ± 3.8	9.1 ± 4.7	*P* = 0.02
	Depression	41	6.1 ± 3.3	10.1 ± 5.6	*P* = 0.03
OD		41	3.1 ± 1.6	4.3 ± 2.2	*P* = 0.005
Motion sickness		41	15.6 ± 4.6	20.7 ± 11.8	*P* = 0.03
ESS		41	9.5 ± 5.8	8.8 ± 6.5	*P* = 0.09

a *All P-values were calculated using the Wilcoxon signed-rank test*.

*DHI, Dizziness Handicap Inventory; HADS, Hospital Anxiety and Depression Scale; OD, Orthostatic dysregulation; ESS, Epworth Sleepiness Scale*.

**Table 4:** Equilibrium test results before and after the earthquake.

**Equilibrium test**		***n***	**Pre**	**Post**	***P-*value**
Romberg ratio	area	41	2.1 ± 1.4	3.2 ± 1.7	*P* = 0.01^a^
	length	41	2.0 ± 1.2	3.7 ± 0.5	*P* = 0.04^a^
AR of cVEMP		41	23.5 ± 13.9 (%)	32.5 ± 19.1 (%)	*P* < 0.001^a^
CP% of caloric test		41	17.3 ± 5.30 (%)	17.9 ± 7.72 (%)	*P* = 0.17^a^
Positive rate of HUT test		41	35.2 ± 11.1 (%)	70.0 ± 10.2 (%)	*P* = 0.03^b^
Positive rate of nystagmus		41	17.9 ± 6.1 (%)	69.2 ± 7.3 (%)	*P* < 0.001^b^

a *P-values were calculated using the Wilcoxon signed-rank test*.

b *P-values were calculated using Fisher's exact test*.

*AR, Asymmetry ratio; cVEMP, cervical vestibular-evoked myogenic potential; CP%, Canal paresis %; HUT, Head-up tilt*.

## Discussion

This study examined the occurrence and possible mechanisms of ERD after major earthquakes. The results showed that the prevalence of balance disorders increased significantly after the major earthquakes when compared to before the earthquakes. The equilibrium test and questionnaire-based survey results also indicated that vestibular functions, especially otolith and autonomic functions, were deteriorated after the major Kumamoto earthquakes and its repetitive aftershocks. Moreover, these results suggested that psychological factors might be a cause for the increase in the prevalence of balance disorders in the patients enrolled in this study. These findings suggest that exposure to major earthquakes and aftershocks induce post-earthquake balance disorders due to sensory conflicts mediated by vestibular dysfunction, autonomic dysfunction, and/or psychological factors, which is congruent with previous findings (7, 32, 33).

One of the main strengths of this study is the use of objective equilibrium testing. In the 41 patients who underwent pre- and post-earthquake equilibrium testing, stabilometry results indicated that the static equilibrium significantly deteriorated during the post-earthquake period compared to that of the pre-earthquake period. In patients with post-earthquake ERD, I also observed a significant deterioration of cVEMP responses. The prevalence of nystagmus was also higher in patients with post-earthquake ERD than in patients with pre-earthquake ERD. A previous study had also reported that healthy individuals who repeatedly experience aftershocks presented with greater equilibrium dysfunction than those who had rarely experienced aftershocks (6). Overall, the above results suggest that vestibular functions worsen after major earthquakes, which might be due to changes in the inertia of saccular otoliths in response to major earthquakes and aftershocks. Otoliths are structures composed of a gelatinous matrix combined with calcium carbonate, which is found in the endolymphatic viscous fluid of the saccule and utricle of the inner ear. The inertia experienced by otolith organs stimulates hair cells and their afferent nerves during head movements (7, 32, 34). The low frequency (0.1–3.5 Hz) (35) horizontal and vertical linear accelerations induced during earthquakes are detected by the vestibular organs, especially the otolith. Besides, the prevalence of BPPV has been shown to increase after major earthquakes (7, 32) and otolith dysfunction is considered a possible underlying mechanism (36, 37). Furthermore, frequent physical shaking may directly impair the vestibular system. Hence, I believe that major earthquakes may concuss the inner ear leading to saccular otolith dysfunction and otoconial detachment from the otolith macula, resulting in canalithiasis and/or cupulolithiasis (7, 32, 34).

Large earthquakes and the physiological and psychological stress they elicit can worsen cardiovascular function (38, 39), which can result in inner ear dysfunction and tissue damage (i.e., hair cell loss and damage to their afferent nerves) through circulatory disturbances in the inner ear, leading to MD, SDV, BPPV, or VBI (40). The increased prevalence of ERD among patients with new-onset MD, SDV, BPPV, or VBI and among patients with recurrence of previously recorded balance disorders, i.e., MD, BPPV, or VBI, may have been caused by an impaired inner ear circulation following cardiovascular dysfunction (40, 41).

Different studies have reported a strong relationship between autonomic dysfunction and balance disorders (25, 31). Generally, the activity of the parasympathetic nervous system in patients with balance disorders is reduced due to an input imbalance through the left or right vestibular nerves to the Edinger-Westphal nucleus, the parasympathetic pre-ganglionic nucleus connected to the vestibular nucleus and inner ear (42), leading to relatively accelerated function of the sympathetic nerve reflexes following visual, vestibular, and/or proprioceptive stimuli. This manifests as changes in BP, increased heart rate, and abnormal blood flow in the vertebral and basilar artery systems, and by extension, the inner ear (42). I found that the proportion of patients with MD, OH, and VN disorders, which are reported to be strongly related to autonomic dysfunction (19, 42–44), was higher during the post-earthquake period, suggesting a possible relationship between balance disorders and autonomic dysfunction. Moreover, the questionnaire-survey results from the 41 patients with ERD revealed increased post-earthquake OD, and motion sickness, as well as increased rates of positive HUT test results, further highlighting the possible role of autonomic dysfunction in the development of ERD.

I observed that equilibrium dysfunction was augmented by psychological stress, as previously reported (45, 46) in both healthy individuals (47) and patients with vestibular dysfunction (48). I observed an increase in the prevalence of new-onset MD, SDV, BPPV, VBI, and psychogenic vertigo, as well as recurrent MD, BPPV, VBI, and VN, after the earthquake; conditions which have been associated with psychological stress (49). Additionally, patients with MD, SDV, psychogenic dizziness, and VN who were evacuated after the earthquake experienced higher psychological stress. The questionnaire-based survey results obtained from patients with ERD suggest that higher levels of anxiety and depression are associated with ERD, providing further proof of the contribution of psychological factors to ERD.

This study has several limitations. First, the sample size of participants who performed the equilibrium test was small. Moreover, ocular VEMP test and video Hit impulse test were not performed because of the breakdown of the measurement instruments due to this major earthquake. Besides, several participating ENT clinics did not have access to these instruments. Examining equilibrium dysfunction in individuals with ERD will enable identification of the causes of post-earthquake dizziness. Conducting equilibrium tests during the post-earthquake period is necessary to increase the sample size and enhance the study's validity. Second, psychological stress, such as post traumatic stress, was not fully investigated; investigating the impact of post-disaster psychological stress on healthy individuals with ERD would help verify the causes of post-earthquake dizziness. Third, the influence of age and sex was not assessed in this study. Future studies should determine whether age or sex might affect the risk of balance disorder after major earthquakes.

In conclusion, exposure to major earthquakes and aftershocks induced post-earthquake dizziness in a significant percentage of individuals. My results indicate that post-earthquake dizziness may be due to sensory conflicts mediated by vestibular dysfunction, autonomic dysfunction, and/or psychological factors. My findings will facilitate the management of dizziness experienced during or after such high magnitude earthquakes, which occur frequently in Japan. Future studies should assess whether earthquakes with less magnitude may affect human sense of balance.

## Data Availability Statement

The raw data supporting the conclusions of this article will be made available by the authors, without undue reservation.

## Ethics Statement

The studies involving human participants were reviewed and approved by Kumamoto University. The patients/participants provided their written informed consent to participate in this study.

## Author Contributions

TM designed and conceptualized the study, acquired and analyzed the data, and drafted the manuscript for intellectual content.

## Conflict of Interest

The author declares that the research was conducted in the absence of any commercial or financial relationships that could be construed as a potential conflict of interest.

## Abbreviations

ANOVA: analysis of variance
BPPV: benign paroxysmal positional vertigo
cVEMP: cervical vestibular-evoked myogenic potential
DHI: Dizziness Handicap Inventory
ENT: ear, nose, and throat
ERD: earthquake-related dizziness
HADS: Hospital Anxiety and Depression Scale
HUT: head-up tilt
MD: Meniere's disease
OD: orthostatic dysregulation
PEDS: post-earthquake dizziness syndrome
SDV: sudden deafness with vertigo
VBI: vertebrobasilar insufficiency
OH: orthostatic hypotension
VN: vestibular neuritis
CI: confidence interval.
