# Supplementary material for: Vestibular Function After the 2016 Kumamoto Earthquakes: A Retrospective Chart Review
Source: Front Neurol. 2021 Jan 22;11:626613. doi: 10.3389/fneur.2020.626613 (PMC7864085; doi:10.3389/fneur.2020.626613)
Supplement: Supplementary file 1 [file Table_1.DOCX]

Supplementary Material

# Supplementary Tables

**Supplementary Table 1**. Overview of the assessment tools used in this study and their respective scoring systems

|  | **Aim** | **Test** | **Scoring and criteria** |
| --- | --- | --- | --- |
| **Questionnaires** | Degree of vertigo/dizziness | DHI (1) | 100-point scale, 25 items  Sub-grouped into three domains: Physical (P), Emotional (E), Functional (F) |
|  | Anxiety and depression | HADS (2) | Anxiety (A) 21-point scale,  7 questions  Depression (D) 21-point scale,  7 questions |
|  | OD | OD questionnaire (3,4) | 11-point scale, 11 questions |
|  | Motion sickness | Motion sickness scores (5) | 56-point scale, 6 questions |
|  | Sleep disorder | ESS (6) | 24-point scale, 8 questions |
| **Tests** | Postural stability | Stabilometry (7)  RR was defined as the ratio of body sway area or length with eyes closed to same parameters with eyes open | Large RR area or length indicated poor static balance |
|  | Otolith function  (Saccule) | cVEMP (8)  AR was defined as the difference between the large amplitude (AL) and small amplitude (AS) of peak 13 to peak n23 divided by the sum of both amplitudes presented as a percentage, i.e., [(AL-AS) / (AL + AS)] × 100 (%). The normal range of AR was defined as less than 33%. | Small AR indicated good saccule-inferior vestibular nerve function |
|  | Semicircular canal and Otolith function  (Utricle) | Caloric test (9)  Canal paresis% (CP%) was calculated using the following equation:  [(MVS of the right warm (RW)+ MVS of the right warm (RW))-(MVS of the left warm (LW)+ MVS of the left cold (LC))]/(RW+RC+LW+LC)x100(%). The normal range of the CP% was defined as less than 20%. | Small CP% (<20%) indicated normal utricle-superior vestibular nerve function |
|  | OD | HUT test (10,11)  The HUT test was performed according to the method established by the Japan Society of Neurovegetative Research in 2015 (29). Non-invasive oscillatory measurements of blood pressure (BP) and pulse rate were performed four times using an automated sphygmomanometer (ES-H55P; Terumo, Tokyo, Japan) at the following time-points: (1) after 10 min in a supine position, (2) after 10 s of standing, (3) after 1 min of standing, and (4) after 10 min of standing (29). The cuff of the BP-recording device was attached to the left arm, which was supported at the heart level throughout the study. The testing was conducted during the daytime in a quiet environment at a constant room temperature of 22–25 °C to exclude the effects of chronobiologic factors on the outcomes of the test. The participants maintained a regular meal schedule but were restricted from smoking and caffeine ingestion for 6 hours before the examination. The intake of foods and medications with sympathomimetic activity was also prohibited before the study. The results were determined as positive or negative according to the outcome of the HUT test and the international scientific definition of OD. | Results of the test were considered positive when meeting either of the following criteria was met:  BP drop ≥ 20/10 mmHg 10 s, 1 min, or 10 min after standing; HR ≥120/min or an increase of 30 beats/min over that at the supine position 10 s, 1 min, or 10 min after standing |
|  | VOR | Nystagmus  Nystagmus was evaluated by the infrared CCD camera. When pathologic nystagmus (i.e. spontaneous nystagmus or positional nystagmus) was observed, the test result was considered positive. | Positive if any nystagmus was observed |

Abbreviations: DHI: Dizziness Handicap Inventory; HADS: Hospital Anxiety and Depression Scale; OD: orthostatic dysregulation; ESS: Epworth Sleepiness Scale; RR: Romberg ratios; AR, asymmetry ratio; cVEMP: cervical vestibular-evoked myogenic potential; CP%, canal paresis %; MVS: maximum slow-phase velocity speed; HUT: head-up tilt; VOR: vestibulo-ocular reflex

**Supplementary Table 2**. Frequency of earthquake-related dizziness according to sex and age at different time-points relative to the time of the earthquake in weeks (w)

|  | **Male (n)** | | | | | | | | | | **Female (n)** | | | | | | | | | |
| --- | --- | --- | --- | --- | --- | --- | --- | --- | --- | --- | --- | --- | --- | --- | --- | --- | --- | --- | --- | --- |
|  | **10s** | **20s** | **30s** | **40s** | **50s** | **60s** | **70s** | **80s** | **90s** | **Total** | **10s** | **20s** | **30s** | **40s** | **50s** | **60s** | **70s** | **80s** | **90s** | **Total** |
| **2 w prior** | 0 | 0 | 1 | 1 | 1 | 3 | 3 | 3 | 0 | 12 | 1 | 2 | 0 | 0 | 2 | 5 | 8 | 6 | 0 | 24 |
| **After 2 w** | 0 | 5 | 3 | 4 | 0 | 3 | 5 | 1 | 0 | 23 | 2 | 1 | 10 | 12 | 23 | 19 | 9 | 5 | 0 | 80 |
| **After 2–4 w** | 1 | 2 | 1 | 0 | 5 | 11 | 5 | 3 | 2 | 30 | 2 | 8 | 12 | 15 | 14 | 17 | 9 | 11 | 2 | 88 |
| **After 4–6 w** | 1 | 1 | 3 | 1 | 7 | 3 | 4 | 5 | 0 | 26 | 2 | 3 | 5 | 7 | 10 | 13 | 17 | 10 | 2 | 69 |
| **After 6–8 w** | 0 | 0 | 1 | 2 | 3 | 4 | 2 | 3 | 0 | 15 | 0 | 0 | 8 | 7 | 8 | 12 | 9 | 5 | 2 | 51 |
| **After 8–10 w** | 0 | 1 | 1 | 3 | 4 | 9 | 8 | 1 | 0 | 27 | 3 | 7 | 8 | 6 | 11 | 10 | 9 | 8 | 0 | 62 |
| **After 10–12 w** | 0 | 1 | 1 | 3 | 5 | 3 | 3 | 2 | 0 | 18 | 1 | 4 | 5 | 7 | 8 | 13 | 6 | 6 | 0 | 50 |

**Supplementary Table 3**. Frequency of earthquake-related dizziness according to the symptoms at different time-points relative to the time of the earthquake in weeks (w)

|  | **The sensation of ground shaking (n)** | **The sensation of lateral vibration (n)** | **Vertigo (n)** | **Light-headedness (n)** |
| --- | --- | --- | --- | --- |
| **2 w prior** | 2 | 14 | 17 | 2 |
| **After 2 w** | 6 | 37 | 40 | 2 |
| **After 2–4 w** | 14 | 40 | 44 | 3 |
| **After 4–6 w** | 4 | 26 | 40 | 7 |
| **After 6–8 w** | 4 | 18 | 37 | 1 |
| **After 8–10 w** | 4 | 31 | 38 | 3 |
| **After 10–12 w** | 5 | 19 | 33 | 2 |

**Supplementary Table 4**. Frequency of earthquake-related dizziness according to the location at different time-points relative to the time of the earthquake in weeks (w)

|  | **Indoor (n)** | **Outdoor (n)** |
| --- | --- | --- |
| **2 w prior** | 2 | 2 |
| **After 2 w** | 13 | 2 |
| **After 2–4 w** | 14 | 0 |
| **After 4–6 w** | 16 | 2 |
| **After 6–8 w** | 6 | 3 |
| **After 8–10 w** | 10 | 0 |
| **After 10–12 w** | 6 | 1 |

**Supplementary Table 5**. Complete list of balance disorders diagnosed by ear-nose-throat doctors in this cohort

| **Diagnosis** | **Number of patients: 575** |
| --- | --- |
| Meniere’s disease | 68 |
| Sudden deafness with vertigo | 41 |
| Benign paroxysmal positional vertigo | 155 |
| Vertebrobasilar insufficiency | 22 |
| Orthostatic hypotension | 48 |
| Psychogenic vertigo | 48 |
| Vestibular neuritis | 16 |
| Perilymph fistula | 1 |
| Hunt’s syndrome | 1 |
| Cervical vertigo | 1 |
| Vertigo of central origin | 4 |
| Motion sickness(including Mal de debarquement syndrome) | 2 |
| Epilepsy | 3 |
| Bilateral vestibulopathy | 0 |
| Others (unknown) | 165 |

# Supplementary Figure 1. Bland-Altman analysis

Bland-Altman analysis with test-retest intervals was performed to discern test-retest effects of clinical changes. (a) DHI, DHI-P, DHI-E, DHI-F (b) HADS-A, HADS-D, (c) OD, (d) Motion sickness, (e) ESS, (f) RR-area, RR-length, (g) Asymmetry ratio calculated from cVEMP amplitude, (h) CP% calculated from Caloric test.

Abbreviations: DHI: Dizziness Handicap Inventory; DHI-P: Dizziness Handicap Inventory- Physical; DHI-E: Dizziness Handicap Inventory- Emotional; DHI-F: Dizziness Handicap Inventory-Functional; HADS-A: Hospital Anxiety and Depression Scale-Anxiety; HADS-D: Hospital Anxiety and Depression Scale-Depression; OD: orthostatic dysregulation; ESS: Epworth Sleepiness Scale; RR: Romberg ratios; cVEMP: cervical vestibular-evoked myogenic potential; CP%, canal paresis percentage

# References

1. Jacobson GP, Newman CW. The development of the Dizziness Handicap Inventory. *Arch Otolaryngol Head Neck Surg* (1990) **116**:424–7.

2. Zigmond AS, Snaith RP. The hospital anxiety and depression scale. *Acta Psychiatr Scand* (1983) **67**:361–70.

3. Okuni M. Orthostatic dysregulation in childhood with special reference to the standing electrocardiogram : Conference on Neurocirculatory Asthenia & Allied Diseases and Orthostatic Hypotensive Diseases. *Jpn Circ J* (1963) **27**:200–4. doi:10.1253/jcj.27.200

4. Tanaka H, Fujita Y, Takenaka Y, Kajiwara S, Masutani S, Ishizaki Y, et al. Japanese clinical guidelines for juvenile orthostatic dysregulation version 1. *Pediatr Int* (2009) **51**:169–79. doi:10.1111/j.1442-200X.2008.02783.x

5. Graybiel A, Wood CD, Miller EF, Cramer DB. Diagnostic criteria for grading the severity of acute motion sickness. *Aerosp Med* (1968) **39**:453–5.

6. Takegami M, Suzukamo Y, Wakita T, Noguchi H, Chin K, Kadotani H, et al. Development of a Japanese version of the Epworth Sleepiness Scale (JESS) based on Item Response Theory. *Sleep Med* (2009) **10**:556–65. doi:10.1016/j.sleep.2008.04.015

7. Tjernström F, Björklund M, Malmström E-M. Romberg ratio in quiet stance posturography—Test to retest reliability. *Gait Posture* (2015) **42**:27–31. doi:10.1016/j.gaitpost.2014.12.007

8. Murofushi T, Kaga K. *Vestibular evoked myogenic potential : its basics and clinical applications.* Tokyo: Springer Japan (2009). doi:10.1007/978-4-431-85908-6

9. Jongkees LBW, Maas JPM, Philipszoon AJ. Clinical nystagmography. *ORL* (1962) **24**:65–93. doi:10.1159/000274383

10. Schellong F, Lüderitz B. *Regulationsprüfung des Kreislaufs funktionelle Differentialdiagnose von Herz- und Gefässstörungen,*. Berlin: Steinkopff (1954).

11. Fedorowski A, Melander O. Syndromes of orthostatic intolerance: a hidden danger. *J Intern Med* (2013) **273**:322–35. doi:10.1111/joim.12021

0
