# Supplementary figures and images for: Vestibular Function After the 2016 Kumamoto Earthquakes: A Retrospective Chart Review
Source: Front Neurol. 2021 Jan 22;11:626613. doi: 10.3389/fneur.2020.626613 (PMC7864085; doi:10.3389/fneur.2020.626613)

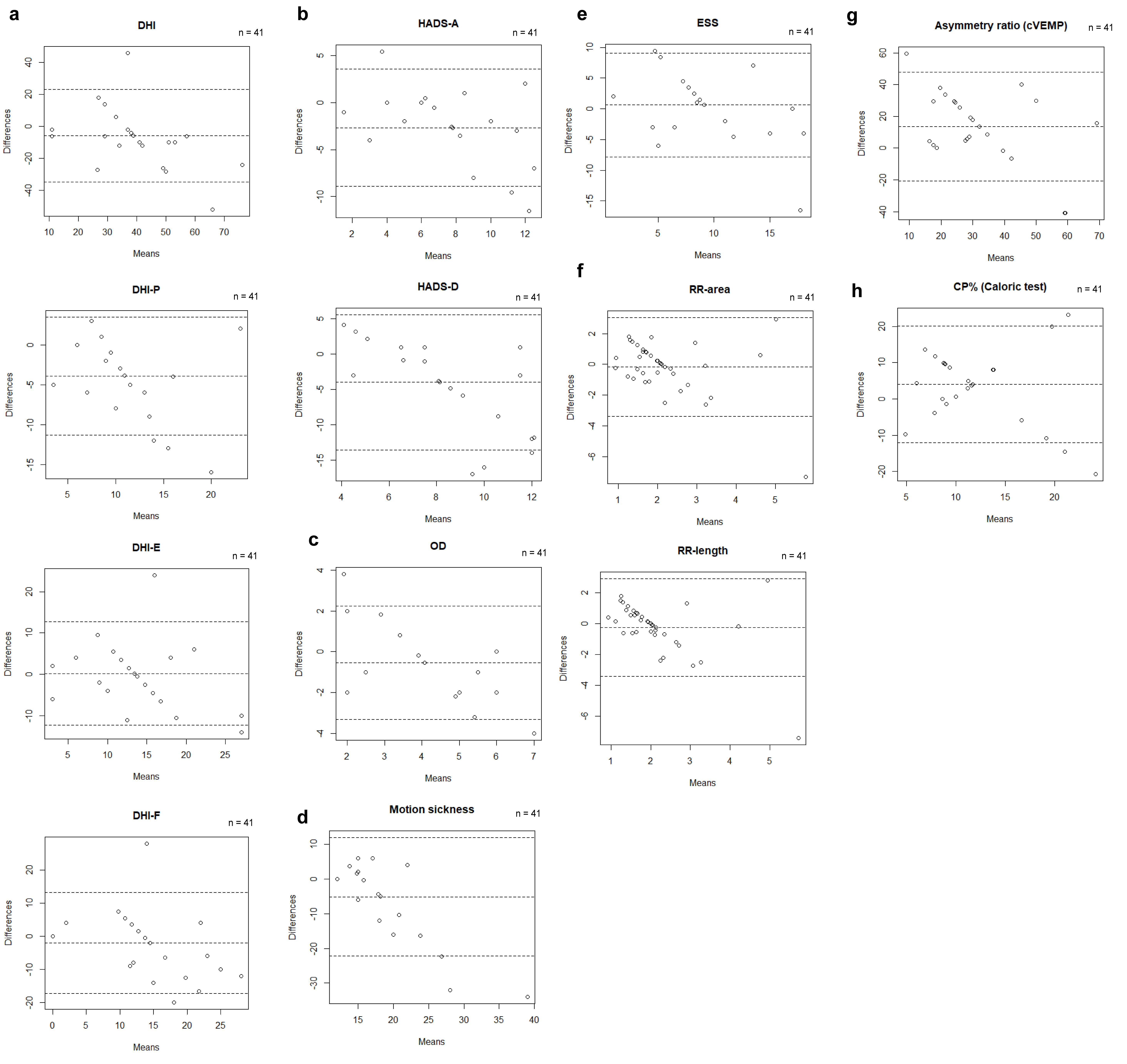

Supplement: Supplementary file 2 [file Image_1.TIF]
